# Growth Analysis of Glyphosate‐Resistant and Susceptible *Amaranthus palmeri* Biotypes

**DOI:** 10.1002/pei3.70023

**Published:** 2024-12-18

**Authors:** Juliana de Souza Rodrigues, Nicholas T. Basinger, Ramon G. Leon, Allan L. Bacha, Renata Thaysa da Silva Santos, Kayla M. Eason, Donn Shilling, Timothy L. Grey

**Affiliations:** ^1^ Department of Crop and Soil Sciences University of Georgia Tifton Georgia USA; ^2^ Department of Crop and Soil Sciences University of Georgia Athens Georgia USA; ^3^ Department of Crop and Soil Sciences North Carolina State University Raleigh North Carolina USA; ^4^ Department of Biology Applied to Agriculture Sao Paulo State University Jaboticabal Sao Paulo Brazil; ^5^ Department of Wood Technology Para State University Maraba Para Brazil; ^6^ Southeast Watershed Research USDA ARS Tifton Georgia USA

**Keywords:** chlorophyll content, leaf area duration, leaf area index, leaf area ratio, leaf weight ratio, net assimilation rate, palmer amaranth, relative growth rate, specific leaf area, stem‐to‐leaf ratio

## Abstract

This study examined the growth parameters of both glyphosate‐susceptible and glyphosate‐resistant biotypes of *Amaranthus palmeri* , designated as GA2005 and GA2017, respectively. A two‐year microplot field study was conducted to assess their growth characteristics. Scheduled destructive harvests on named harvest days (HD) were conducted to collect measurements for further calculation of net assimilation rate (NAR; g m^−2^ day^−1^), specific leaf area (SLA), leaf weight ratio (LWR), stem‐to‐leaf ratio (SLR), leaf area index (LAI), leaf area ratio (LAR; cm^2^ g^−1^), leaf area duration (LAD; days), relative growth rate (RGR; g.g^−1^ day^−1^) and plant volume (m^3^). In addition, stem diameter, number of leaves, and Chlorophyll content (μmol m^2^) were determined. The main objective was to identify growth parameters that differentiate biotypes along the plant life cycle. While certain growth parameters showed no variation among biotypes, differences in leaf area index (LAI) over HD and chlorophyll content and leaf area duration (LAD) were observed as the main effects. Glyphosate‐resistant biotypes exhibited higher LAD and chlorophyll content, potentially conferring a competitive advantage, especially in heavily used glyphosate environments. The study highlights the complexity of intraspecific genetic differentiation, adaptation, and environmental factors affecting *A. palmeri.* It may offer insights into biotype distinction and resistance spread while advancing our comprehension of species adaptation and growth strategies for enhanced control.

## Graphical abstract

The growth patterns of weed populations across diverse geographical regions could impact the growth and development disparities between Palmer amaranth biotypes, revealing insights into their intraspecific growth strategies. Notably, leaf area index (LAI), leaf area duration (LAD), and chlorophyll content (μmol m^2^) emerged as the most distinctive parameters during the evaluation of glyphosate‐susceptible and resistant biotypes.

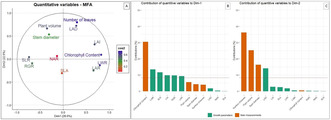

## 1 Introduction

The agricultural sector has witnessed significant advancements over the past 70 years with the introduction of herbicides, resulting in improved weed management and increased crop yields (Shaner 2014). With the continuous progress of technology and the introduction of new herbicides and mechanisms of action over time, the emergence of herbicide‐resistant crops has led to increased use of these chemicals (Duke and Powles 2008). However, the extensive use of chemical weed control comes at a price: weeds adapt at a rapid pace (Landau et al. 2023). A growing number of reports indicate the survival of plants after herbicide applications, posing a significant challenge to modern agriculture (Neve, Vila‐Aiub, and Roux 2009).

*Amaranthus palmeri S. Watson* is a diecious summer annual specie that has been considered one of the most troublesome weed species regarding herbicide resistance in several countries (Roberts and Florentine 2021). According to Heap (2024), resistance has been reported in the following herbicide groups: acetolactate synthase inhibitors (ALS, group 2), microtubule assembly inhibitors (group 3), photosystem II inhibitors (groups 5 and 6), enolpyruvyl shikimate phosphate synthase inhibitors (EPSP, group 9), protoporphyrinogen oxidase inhibitors (PPO, group 14), glutamine synthase inhibitor (group 10), hydroxyphenyl pyruvate dioxygenase inhibitor (HPPD, group 27), auxin mimics (group 4), and very long‐chain fatty acid synthesis inhibitors (VLCFA, group 15).

In Georgia, the first reported case of *A. palmeri* resistant to glyphosate was reported by Culpepper et al. (2008) in 2005 and multiple resistance was reported in 2010 in corn fields (Heap 2024). Glyphosate became an important tool for weed control following the release of glyphosate‐resistant cotton varieties in 2001 (Webster and Nichols 2012). *A. palmeri* is characterized by its remarkable survival capacity in various harsh environments, early‐season emergence, ability to produce a significant volume of seeds, and rapid growth rate (Mahoney et al. 2021; Webster and Grey 2015; Ward, Webster, and Steckel 2013; Culpepper et al. 2008).

Populations of *A. palmeri* exhibit high genetic variability within populations and lower variability between geographically separated populations when assessed using neutral genomic markers (Chandi, Milla‐Lewis, and Jordan 2013) and Q_ST_ − F_ST_ analysis (Leon and van der Laat 2021). As a result, establishing a uniform population is challenging and requires intense selection to fix specific traits (Leon, Dunne, and Gould 2021). Furthermore, while neutral genomic markers are typically not influenced by selection forces, the selective pressures in agricultural fields can be significant enough to override gene flow. This can lead to the fixation of alleles or traits that enhance the weed's survival in specific cropping systems (Karn and Jasieniuk 2017).

Intraspecific variation regarding functional traits arises from inheritable genetic diversity and phenotypic adaptability (Whitham et al. 2020). This diversity significantly impacts how plants respond to various environmental and biological factors, affecting their morphology, (eco)‐physiology, and reproduction (Siefert et al. 2015; Bajwa, Chauhan, and Adkins 2017). Intraspecific trait variation is often mentioned as a critical tool for understanding variation at the individual level, reflecting on the plant‐environmental interaction response (Siefert et al. 2015).

Pertaining to phenotypic adaptability, the analysis of growth parameters is a crucial tool in assessing weed adaptability and provides an in‐depth understanding of weeds' tactics to thrive in diverse conditions (Horak and Loughin 2000). Comparisons of leaf area, height, plant volume, and dry weight offer insights into the species' relative size, productivity, and photosynthetic capacity, which could impact its competitive ability (Radosevich, Holt, and Ghersa 1997). Calculated growth parameters such as specific leaf area (SLA), leaf weight ratio (LWR), and leaf area ratio (LAR) were used to estimate the photosynthetic area per biomass unit. Comprehending these intraspecific differences is essential for identifying life‐history traits that enhance competitiveness, pivotal in plant evolution (Jasieniuk, Brûlé‐Babel, and Morrison 1996).

Thus, this study aims to compare the growth parameters of an *A. palmeri* biotype prior to the spread of glyphosate resistance to a resistant biotype from the same location as the first reported resistance case in Georgia. The objective is to elucidate the growth and development variations between the two *A. palmeri* biotypes and to identify plant growth parameters relevant to this comparison. This will establish a foundation for more in‐depth and expandable comparisons in the future.

## 2 Materials and Methods

### 2.1 Plant Material

*A. palmeri* seeds, GA2005 and GA2017, previously evaluated for herbicide resistance, with confirmed glyphosate resistance (ED50's: 0.272 a.e. kg ha^−1^ and 1.180 a.e. kg ha^−1^, respectively) (de Souza Rodrigues et al. 2023) were collected at Tift (susceptible population) and Macon‐Bibb (resistant population) counties in GA, US, in 2005 and 2017.

Glyphosate resistance was confirmed by conducting a shikimate assay. *A. palmeri* seeds were sown in 72‐cell trays filled with potting media (Pro‐Mix, BX, Quebec, Canada). The trays were maintained in a greenhouse under a 16/8 h day/night photoperiod, supplemented with light providing 600 μmol m^−2^ s^−1^. Sprinkler irrigation was provided twice daily for 8 min each time. After germination and seedling emergence, plants were thinned to one plant per cell. Seedlings from each population at the 8–10 leaf growth stage were used as tissue sources for subsequent tests. The shikimate assay was conducted using 7 mL scintillation vials, following modified protocols from Shaner et al. (2005) and Hoagland et al. (2013). Ten leaf discs (4 mm) from 3 to 4 plants of each biotype were added to separate vials. Each vial received 1000 μL of 10 mM ammonium phosphate solution with 0.1% (v/v) Tween 80 surfactant and glyphosate at one of the concentrations: 1000, 700, 300, 180, 75, 25, 5, or 0 μM. The vials were gently shaken to ensure complete submersion of the discs and then incubated under light at 200 μmol m^−2^ s^−1^ for 24 h. Post‐incubation, vials were frozen at −20°C until shikimate analysis. The frozen vials were thawed at 60°C for 30 min, after which 250 μL of 1.25 M HCl was added to each vial, followed by another 15‐min incubation at 60°C. From each vial, 25 μL of the HCl extract was transferred to a microtiter plate, where 100 μL of 0.25% (w/v) periodic acid and 0.25% (w/v) m‐periodate solution was added to each well. After incubating at 25°C for 90 min, 100 μL of 0.6 N sodium hydroxide/0.22 M sodium sulfite solution was added, and optical density at 380 nm was measured within 30 min using a microtiter plate spectrophotometer. Background optical density was assessed from control wells containing untreated leaf discs and subtracted from each glyphosate‐treated well. To generate a shikimate standard curve, known concentrations of shikimate (0–360 μM) were added to vials containing 10 untreated leaf discs. Shikimate levels were reported as μg of shikimate per milliliter of HCl solution.

### 2.2 Greenhouse Seedling Establishment and Outdoor Microplot Experiment

**FIGURE 1:**
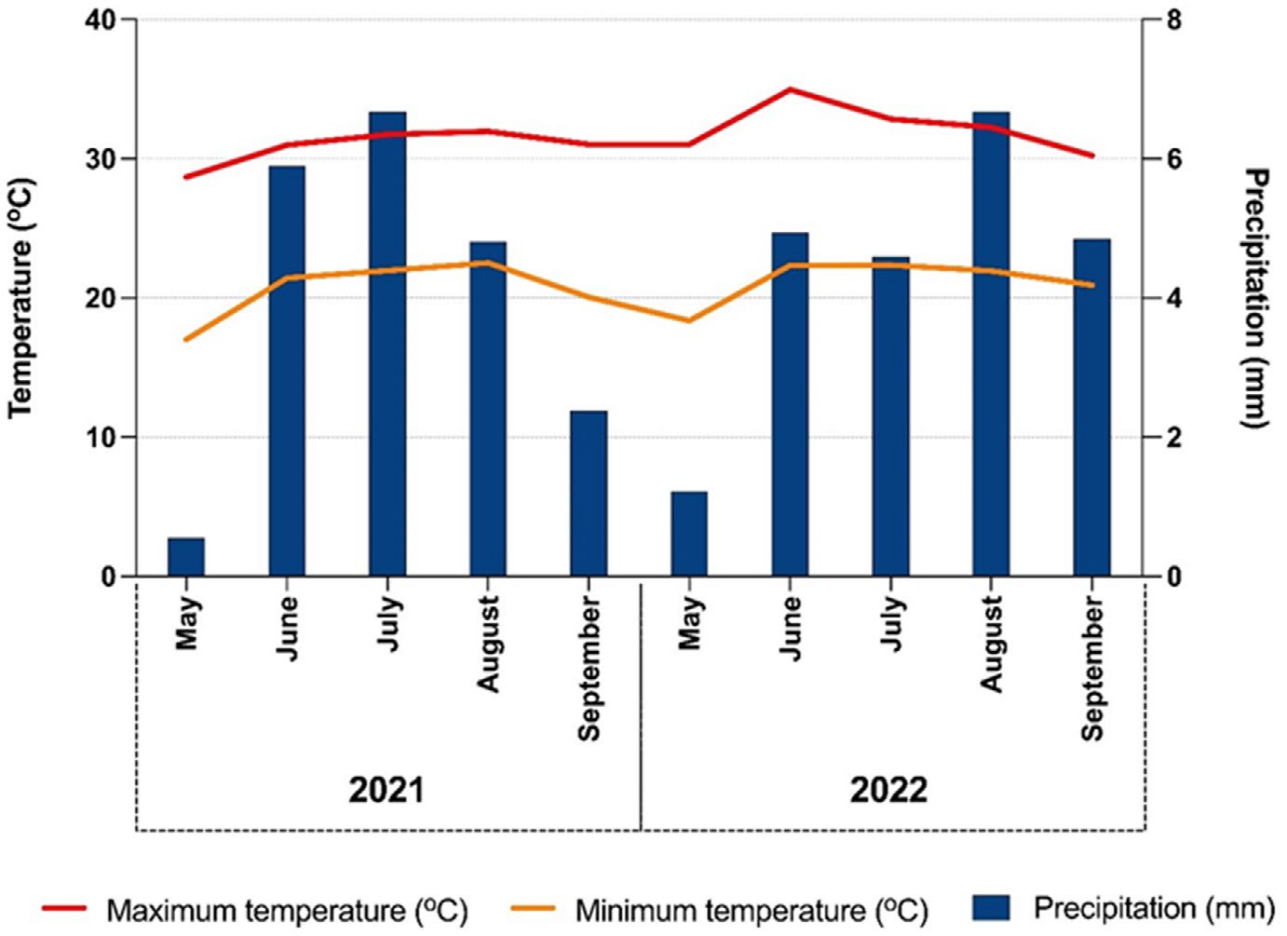
Mean maximum and minimum temperatures (°C) and precipitation (mm) recorded during the growing period (from May to September) for *Amaranthus palmeri* at USDA, Tifton, GA, in 2021 and 2022.

Seeds were planted in trays containing potting media (Pro‐Mix, BX, Quebec, Canada). The trays were placed in a greenhouse with a 16/8 h (day/night) photoperiod, supplemented with light providing 600 μmol m^−2^ s^−1,^ and sprinkler irrigation was scheduled for 15 min, twice a day. Once the seedlings reached 8–10 cm in height, they were transplanted into outdoor microplots (pots with 76 cm diameters buried into the ground to a depth of 76 cm) to avoid variation due to competition with other plants (Webster, Grey, and Ferrell 2017). The pots were filled with Tifton loamy sand (fine‐loamy, kaolinitic, thermic, plinthic, Kandiudult) with pH 6.5 and organic matter 0.8%, located at the U.S. Department of Agriculture–Agricultural Research Service Crop Protection and Management Research Unit in Tifton, GA (31.48° N, 83.53° W), during the growing seasons in 2021 and 2022. Plants were fertilized every week through manual irrigation with 24‐8‐16 (Miracle‐Gro All Purpose Plant Food watering can, Marysville, Ohio) until the last harvest performed. The average maximum and minimum temperatures (°C), as well as rainfall (mm) during the experiment, are shown in Figure 1. Weather data were obtained from the University of Georgia Weather Network for the Tift County station, GA.

### 2.3 Research Design

The experimental design consisted of a randomized complete block with three blocks in a factorial structure of biotypes (GA2005 and GA2017), and five harvest days (*n* = 60 plants). Data from the 2021 and 2022 trials were combined. The study was designed to collect data points between May and September by scheduling multiple harvest days. Six plants were collected at every harvest, three glyphosate‐resistant and three glyphosate‐susceptible. The plants could grow for 30 days after transplant, before the first harvest. Subsequently, the second, third, and fourth harvests were conducted at 14‐day intervals, while the fifth and final harvests occurred 30 days apart (Table 1).

**TABLE 1:** Harvest days (HD) throughout the growing seasons of *Amaranthus palmeri* in 2021 and 2022.

Year	Seedling transplanting date	HD 1	HD 2	HD 3	HD 4	HD 5
		30 DAT	44 DAT	58 DAT	72 DAT	102 DAT
2021	May 21	June 21	July 12	July 26	August 9	September 10
2022	May 24	June 27	July 11	July 25	August 8	September 9

During each harvest, several plant measurements were recorded, including height (cm), diameter (cm), leaf area (cm^2^), number of leaves, and chlorophyll content (μmol m^2^). The plants were cut at the base, divided into leaves, stems, and inflorescences, and placed in separate paper bags. To obtain constant dry matter, the plant parts in the bags were dried in the oven through forced air circulation at 60°C. The leaf area was measured using the LI‐3100C area meter (LI‐COR in Lincoln, NE). The mean of three measurements determined the chlorophyll content (μmol m^2^) on a fully expanded leaf at the plant top on every plant harvested using the MC‐100 chlorophyll meter (Apogee, Logan, UT).

### 2.4 Analysis

All data collected was used to evaluate and compare the growth and development of two biotypes. Plant volume (m^3^) was calculated following the equation proposed by Horak and Loughin (2000) estimating an elliptical column, which is used as a surrogate for plant volume, as:

$$\text{elliptical column}=\text{height}\times \frac{1}{2}\text{diameter}\;1\times \frac{1}{2}\;\text{diameter}\;2\times \pi$$

where, diameter 1 represents the horizontal 180° plant diameter, spanning the widest direction, while diameter 2 denotes the vertical measurement 90° to the widest direction, extending from the top to the soil level.

Then, crop growth indices were calculated to evaluate the development of the biotypes throughout the growing season. The calculations were performed according to the equations given in Gardner, Pearce, and Mitchell (1985), Hunt (1990) and Hunt et al. (2002) including leaf area index (LAI, ratio of green leaf area/ground area); leaf area ratio (LAR, ratio of the total leaf area to the total above‐ground dry weight, cm^2^ g^−1^); leaf weight ratio (LWR, express the dry weight of leaves to whole plant dry weight, g g^−1^); specific leaf area (SLA, leaf area produced per unit leaf dry matter, m^2^ g^−1^); relative growth rate (RGR, dry weight increase in a time interval related to the initial weight, g g^−1^ day^−1^); net assimilation rate (NAR, dry matter increment over time based on leaf area, g m^−2^ day^−1^), stem to leaf ratio (SLR, ratio of stem dry matter to leaf dry matter, g g^−1^), and leaf area duration (LAD, e duration and extent of photosynthetic tissue of the plant, days). The calculations were performed to obtain the growth indices at every harvest interval.

### 2.5 Statistical Analysis

Data recorded from the measurements obtained at every harvest day were used to calculate LAI, LAR, LWR, SLA, RGR, NAR, SLR, LAD, and plant volume (m^3^). In addition, stem diameter (cm), number of leaves, and chlorophyll content (μmol m^2^) were recorded at every harvest interval. However, since they were not used in the growth parameter calculations, the variables proceeded for further analysis. Shapiro–Wilk and Levene's test were carried out to identify any non‐normality and non‐constant variance in each response variable evaluated. Corrective log transformations were applied as necessary.

A multiple factor analysis (MFA) was performed to identify which variables collected were responsible for the most variability on this dataset. The choice of method was based on the criteria proposed by Nguyen and Holmes (2019). Kaiser–Meyer–Olkin (KMO) and Bartletts' tests were performed to evaluate if the data was suitable for factor analysis. Variables were separated into groups for analysis, such as treatment (categorical; biotype, harvest days and year), group I (main measurements: number of leaves, stem diameter (cm), chlorophyll content (μmol m^2^), and plant volume (m^3^)), and group II (calculated growth parameters: LAR, SLA, LAI, LWR, RGR, NAR, SLR, and LAD). Five dimensions were fixed by default, and the eigenvalues were obtained and compared to the other values in those dimensions that were also significant, according to a *p* < 0.05. We tested the importance of correlation with MFA dimensions Dim 1 and Dim 2, cos2 values, eigenvalues, and the significance of the different variables on MFA dimensions 1 and 2. ‘FactoMineR’ package (Lê, Josse, and Husson 2008) and ‘factoextra’ (Kassambara 2016) were used to extract and visualize the results on RStudio Core Team (2022). Finally, a plot of these values was created to visualize the relationships.

The MFA, as explained by Abdi, Williams, and Valentin (2013), is a method used in multivariate data analysis. It is particularly suitable for datasets where observations are described by multiple sets of variables. One of MFA's primary goals is to bring out strong patterns from these sets while considering their structured nature. This is achieved by normalizing the datasets to create a shared space or a typical representation of the observations. Essentially, the common representation extracted via MFA is somewhat similar to the output of Principal Component Analysis (PCA), where the total variance of the data is broken down into orthogonal dimensions known as principal components. However, the difference lies in how MFA gives a balanced representation of each group of variables, ensuring that no group dominates the overall variability due to its inherent scale or variance. For a more detailed and step‐by‐step overview of the MFA process, Pagès (2014) provides a comprehensive guide.

Data selected post‐MFA analysis was analyzed with a mixed effects ANOVA (α = 0.05). Biotypes and Harvest Days (HD) were considered fixed effects, and the year and blocks were random. When normality and homoscedasticity assumptions were not respected, data were transformed in the log prior to analysis. Post hoc tests were applied to transformed data, but non‐transformed data means were presented. A set of contrasts evaluated significant interactions. Pairwise contrasts were tested for factors that were not involved in significant interactions but had significant main effects. All pairwise contrasts were tested with the Tukey HSD test at α = 0.05, and mean differences are shown with the compact letter display (CLD) (Piepho 2018). Shikimate accumulation data were subjected to non‐linear regression using a log‐logistic three‐parameter model. The three‐parameter log‐logistic model, with the lower limit being zero:

$$y=\frac{d}{1+\exp(b\left(\log\left(x\right)-\log\left(e\right)\right)}$$

where *d* is the upper asymptote, b is the slope of the curve and e is the inflection point equal to the ED50. Model selection was based on Akaike's information criterion (AIC).

The analyses were carried out in RStudio Core Team (2022), with the lme4 (Bates et al. 2015), nlme (Pinheiro and Bates 2023), lmerTest (Kuznetsova, Brockhoff, and Christensen 2017), emmeans (Lenth 2023), car (Fox and Weisberg 2019), corrplot (Wei and Simko 2021), GGally (Emerson et al. 2013), ggplot2 (Wickham 2016) and drc (Ritz et al. 2015) packages.

## 3 Results

**FIGURE 2:**
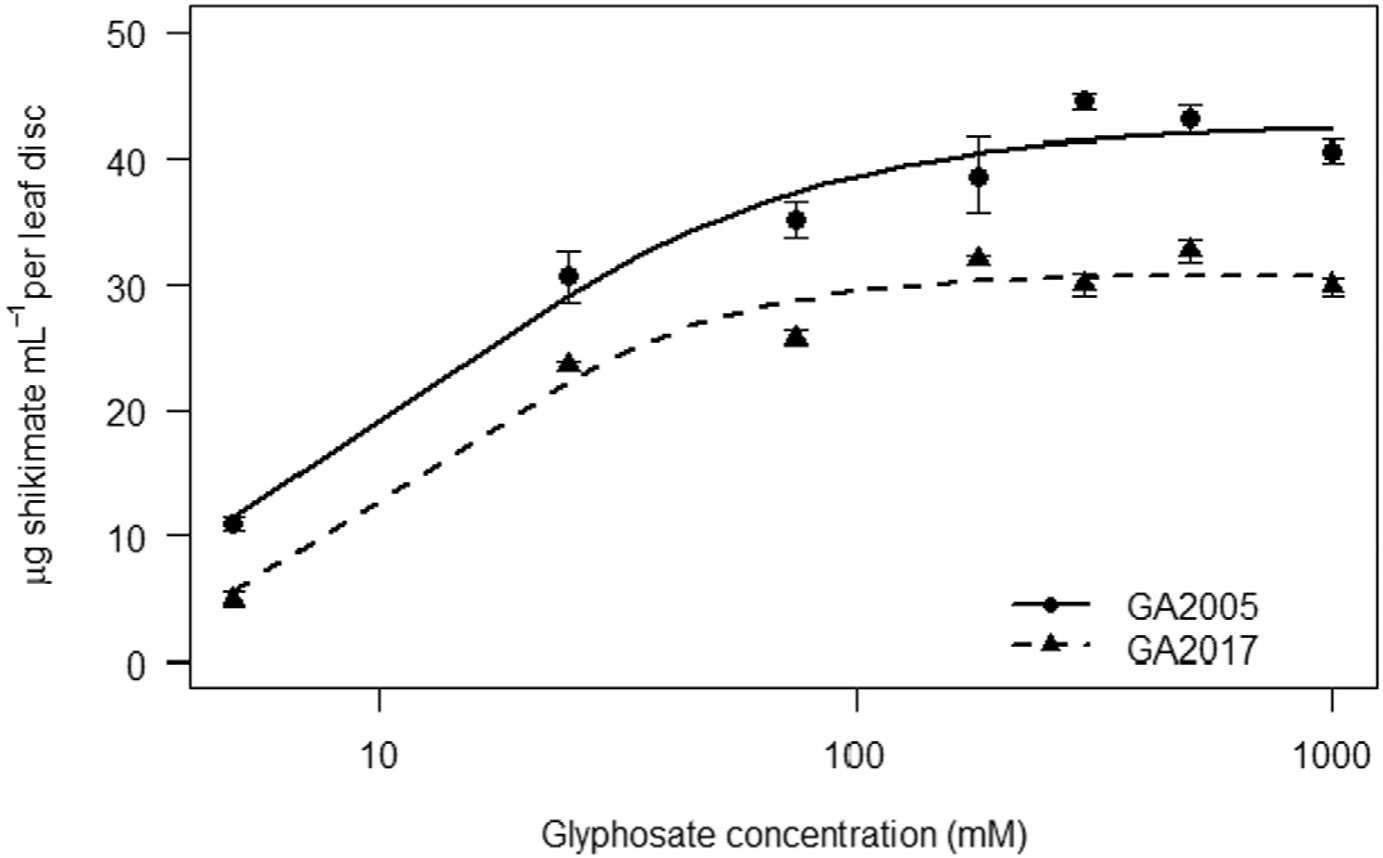
Accumulation of shikimate in leaf discs of three *A. palmeri* biotypes exposed to various concentrations of glyphosate (log_10_ scale). GA2005 (glyphosate‐susceptible), GA2017 (glyphosate‐resistant). Data points are means and standard errors.

The shikimate concentration for the resistant biotype (GA2017) was low at every dose evaluated (Figure 2), compared to the susceptible biotype (GA2005). Shikimate increased in all biotypes as glyphosate concentration increased, but the increase was greater in GA2005.

$$\mathrm{GA}2005:y=\frac{42.63}{1+\exp(-1.08\left(\log\left(x\right)-\log\left(12.37\right)\right)}$$

$$\mathrm{GA}2017:y=\frac{30.85}{1+\exp(-1.53\left(\log\left(x\right)-\log\left(15.37\right)\right)}$$

The cutoff for eigenvalues was selected based on the Kaiser criteria (Kaiser 1960), considering values larger than 1. This led to the choice of two dimensions (Table 2) that explained 51.5% of the data variation together. A cutoff of 0.4 for cos2 was applied for quantitative variable contribution, as per Stevens (1992), leading to the further analysis of chlorophyll content, stem diameter, number of leaves, plant volume, LAR, LAI, LWR, RGR, SLR, and LAD. Due to low contributions (cos2 < 0.4), SLA and NAR were not used for further analysis (Table 2).

**TABLE 2:** Group contributions (%), cos^2^ ^a^ values, significant correlations (*p* < 0.05), and eigenvalues indicating the importance of the measured variables on dimensions 1 (Dim 1) and 2 (Dim 2) of the Multiple Factor Analysis for the growth parameters evaluated on *Amaranthus palmeri* biotypes, glyphosate‐susceptible (GA2005) and glyphosate‐resistant (GA2017).

Groups	Dim 1		Dim 2	
	Contributions (%)			
Main measurements	44.11		78.17	
Growth parameters	55.89		21.83	

Quantitative variables	Dim 1		Dim 2	
	Cos^2^	*R* ^2^	Cos^2^	*R* ^2^
Stem diameter (cm)	0.09	−0.30	**0.40**	0.53***
Number of leaves	0.09	0.30	**0.63**	0.79***
Chlorophyll content (μmol m^2^)	**0.67**	0.82***	0.01	—
Leaf area ratio (LAR)	**0.43**	0.65***	0.06	—
Specific leaf area (SLA)	0.01	−0.11	0.09	−0.30
Leaf area index (LAI)	**0.46**	0.71***	0.10	0.31
Leaf weight ratio (LWR)	**0.62**	0.79***	0.01	—
Relative growth rate (RGR)	**0.45**	−0.67***	0.00	—
Net assimilation rate (NAR)	0.02	−0.11	0.00	—
Stem to leaf ratio (SLR)	**0.54**	−0.74***	0.00	—
Leaf Area Duration (LAD)	0.08	0.28	**0.52**	0.72***
Plant volume (m^3^)	0.12	−0.35	**0.44**	0.66***
Eigenvalues^b^ (variance)	1.4		1.1	
Eigenvalues^b^ (% of variance)	28.5		22.6	

*Note: R* ^2^: correlation of each variable with the respective dimension. Significant data used for further analysis are in bold. Significace code: ‘***’ 0.001.

^a^ Cos^2^ (square cosine): quality of variable representation on factor map; values > 0.4 were considered.

^b^ Eigenvalues: the amount of variation retained by each dimension. Values > 1 are considered a cut off, representing factors with more variance in the standardized data. Together, Dim 1 and Dim 2 account for 51.5% of variance.

**FIGURE 3:**
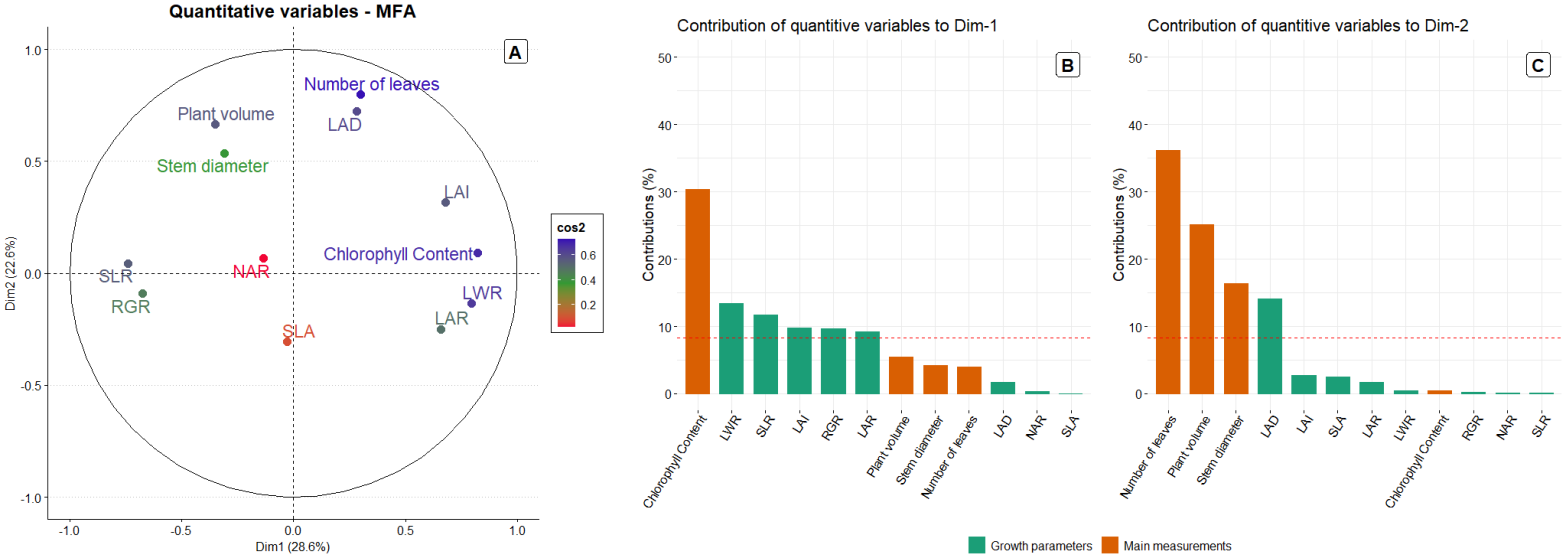
(A) Factor map with the respective quality of representation (cos^2^) of the variables (number of leaves, stem diameter (cm), and chlorophyll content (μmol m^2^), leaf area index (LAI), leaf area ratio (LAR), leaf weight ratio (LWR), relative growth rate (RGR, g g^−1^ day^−1^), net assimilation rate (NAR, g m^−2^ day^−1^), stem to leaf ratio (SLR), specific leaf area (SLA, m^2^ g^*−*1^), leaf area duration (LAD, days), and plant volume (m^3^)) studied for GA2005 and GA2017, *Amaranthus palmeri* biotypes. (B and C) Percent contribution of growth parameters and main measurements to Dim 1 and 2, respectively, of the MFA. The red dotted line represents the expected average contribution of the analyzed factors.

Variable representation for the assessed factors is depicted on the factor map (Figure 2), where the cos^2^ assesses representation quality on the plot. Variables with vectors distant from the origin show a good representation on this plot, validating the cos^2^ values and correlations discovered for the studied variables, as shown in Table 2. Chlorophyll content exhibits the highest correlation in dimension 1, followed by LWR, LWR, SLR, LAI, and RGR. In contrast, the higher correlation of the number of leaves, LAD, plant volume, and stem diameter was identified in dimension 2. Chlorophyll content, LWR, SLR, LAI, RGR, and LAR contributed greater than the expected average to DIM1 (Figure 3B), while several leaves, plant volume, stem diameter, and LAD to Dim‐2 (Figure 3C).

To evaluate the differences among the selected variables post‐MFA analysis, ANOVA results are detailed in Table 3. Interestingly, the stem diameter (cm) holds significance in accounting for the variance observed in the MFA analysis (cos^2^ = 0.53) (Table 2). However, the univariate analysis reveals its non‐significance, indicating that this variable by itself is insufficient for comprehending the differences among biotypes harvested over time (HD).

**TABLE 3:** ANOVA table (p‐values) for chlorophyll content (μmol m^−2^), number of leaves, stem diameter (cm), LWR, LAI, RGR, SLR plant volume (m^3^), LAD, LAR for *Amaranthus palmeri* biotypes, glyphosate‐susceptible (GA2005) and glyphosate‐resistant (GA2017), evaluated at 30, 44, 58, 72 and 102 harvest days (HD), and their interaction.

Treatment factors	Biotype	Harvest days (HD)	Biotype × Harvest days (HD)
Chlorophyll content (μmol m^−2^)	0.0418*	ns	ns
Relative growth rate (RGR)	ns	< 0.0001***	ns
Plant volume (m^3^)	ns	< 0.0001***	ns
Number of leaves	ns	< 0.0020**	ns
Stem diameter (cm)	ns	ns	ns
Stem to leaf ratio (SLR)	ns	< 0.0001***	ns
Leaf weight ratio (LWR)	ns	< 0.0001***	ns
Leaf area index (LAI)	0.0096**	< 0.0001***	0.0485*
Leaf area duration (LAD)	0.0156*	< 0.0001***	ns
Leaf area ratio (LAR)	ns	< 0.0001***	ns

*Note:* Significant. codes: ‘***’ 0.001, ‘**’ 0.01, ‘*’ 0.05, ns = non‐significant at *p* = 0.05. Leaf weight ratio (LWR, index of leafiness of the plant on a dry weight basis, g.g^−1^). Leaf area index (LAI, ratio of green leaf area/ground area), Relative growth rate (RGR, dry weight increase in a time interval related to the initial weight, g.g^−1^.days^−1^). Stem to leaf ratio (SLR, ratio of stem dry matter content to leaf dry matter, g.g^−1^). Leaf area duration (LAD, LAI over a period, of days).

**FIGURE 4:**
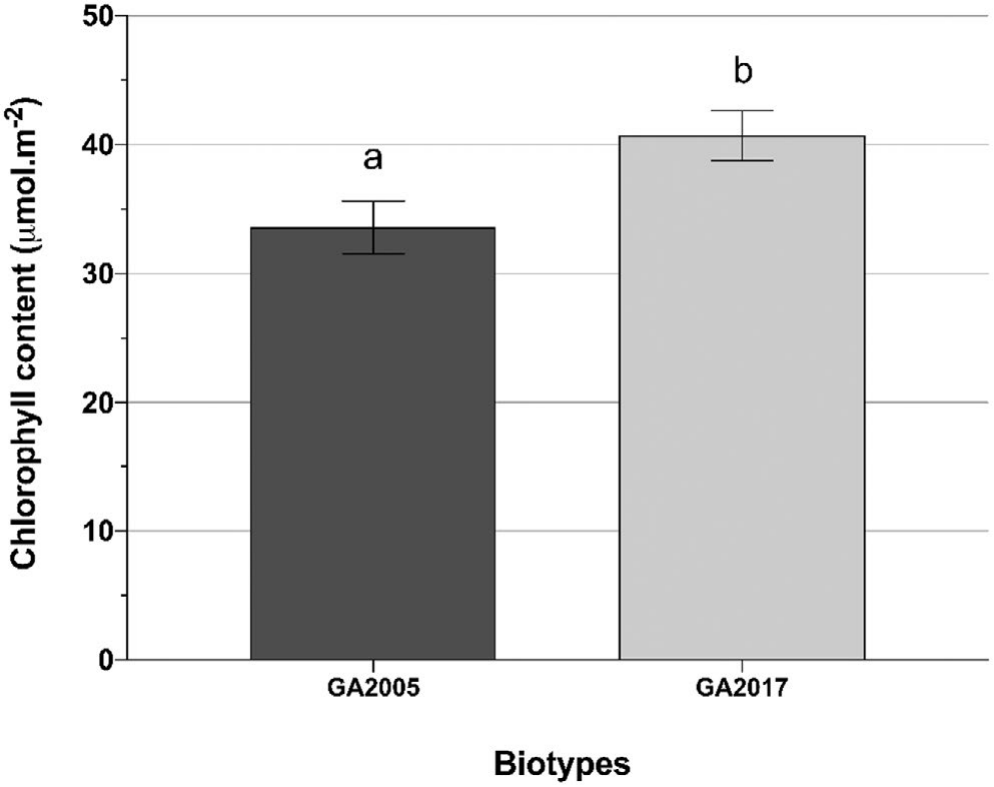
Main effects for chlorophyll content (μmol m^−2^) of *Amaranthus palmeri* biotypes, glyphosate‐susceptible (GA2005) and glyphosate‐resistant (GA2017). Data presented are means ± SE (standard error) from the original data. Means followed by the same letter are not different based on the Tukey HSD test at α = 0.05.

Chlorophyll content was significant (*p* < 0.0418) for biotype. GA2017 had 17% more chlorophyll content than GA2005 (Figure 4).

**FIGURE 5:**
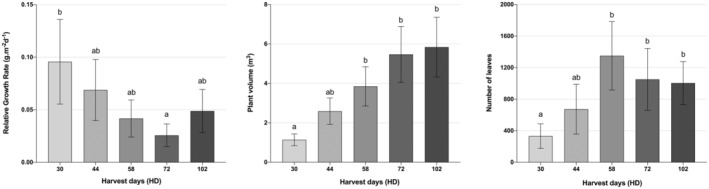
The main effects of relative growth rate (RGR, g.g^−1^d^−1^), plant volume (m^3^), and the number of leaves of *Amaranthus palmeri* biotypes were observed over harvest days (HD). Harvest days (HD) refer to the number of days from *A. palmeri* transplanting to the scheduled harvest, which occurred at 30, 44, 58, 72, and 102 days. Data presented are means ± SE (standard error) from the original data. Means followed by the same letter are not different based on the Tukey HSD test at α = 0.05.

RGR was significant for HD (*p* < 0.001), decreasing over time and indicating reduced vegetative development (Figure 5). Plant volume and the number of leaves increased significantly for HD only (*p* < 0.001, Figure 5). Plant volume achieved 5.84 m^3^ on average at 102 HD. Number of leaves had a peak at 58 HD (1350 leaves), maintaining the average until the last harvest at 102 HD (1004 leaves), (Figure 5). These results are expected since plants' growth, volume, and the number of leaves increases until lower leaves are shaded, leading to a decrease in RGR over time.

**FIGURE 6:**
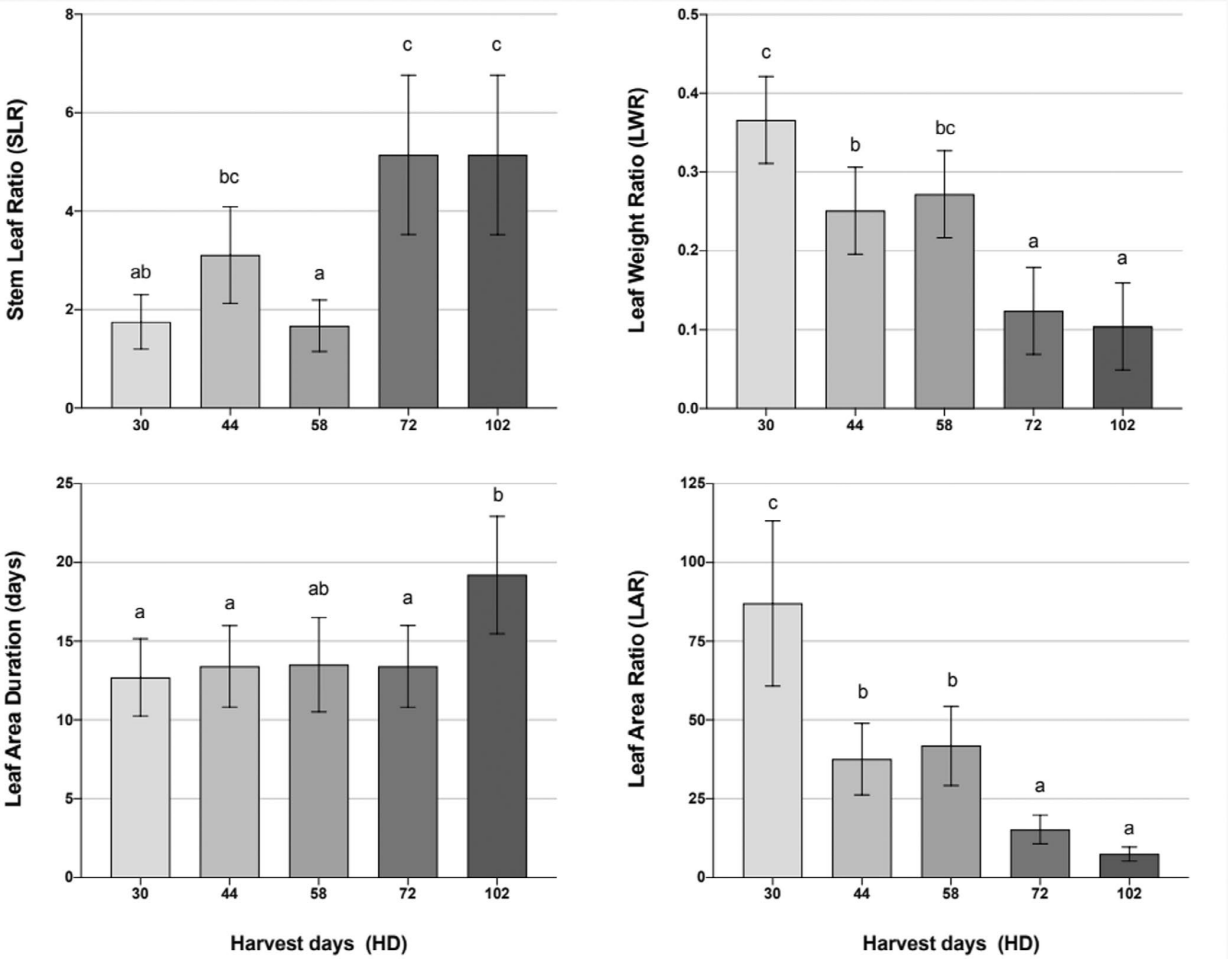
Main effects for stem leaf ratio (SLR), leaf weight ratio (LWR, g.g^−1^), leaf area duration (LAD), and leaf area ratio (LAR) of *Amaranthus palmeri* biotypes observed over harvest days (HD). Harvest days (HD) refer to the number of days from *A. palmeri* transplanting to the scheduled harvest, which occurred at 30, 44, 58, 72, and 102 days. Data presented are means ± SE (standard error) from the original data. Means followed by the same letter are not different based on the Tukey HSD test at α = 0.05.

The stem‐to‐leaf ratio (SLR) represents the dry matter distribution between the stem and leaves during growth. Significant variation in SLR (*p* < 0.001) was observed over HD (Table 3). Due to the first harvest occurring at 30 HD, *A. palmeri* was relocating growth to stem and/or reproductive structures since SLR ≥ 1 (Figure 6). More resources could have been allocated to leaves to allow light interception and subsequent growth at the beginning of the growth period. In addition, investment in stem growth is an advantageous strategy during growth since plants can outcompete neighbors for light more efficiently.

LWR and LAR exhibited statistical significance (*p* < 0.001) across harvest days (HD) (Table 3). LWR, which represents the fraction of total plant weight allocated to leaves, demonstrated a declining trend from 0.336 g.g^−1^ at 30 HD to 0.104 g.g^−1^ at 102 HD (Figure 6). In addition, LAR, which reflects the size of the assimilatory apparatus, decreased over time, declining by 91% by 102 HD as *A. palmeri* plants matured (Figure 6). The rapid early growth phase suggested that after 44 HD, a significant portion of photosynthates were allocated to reproductive and root growth. Higher LAR values may confer greater photosynthetic capacity, potentially providing a competitive advantage.

**FIGURE 7:**
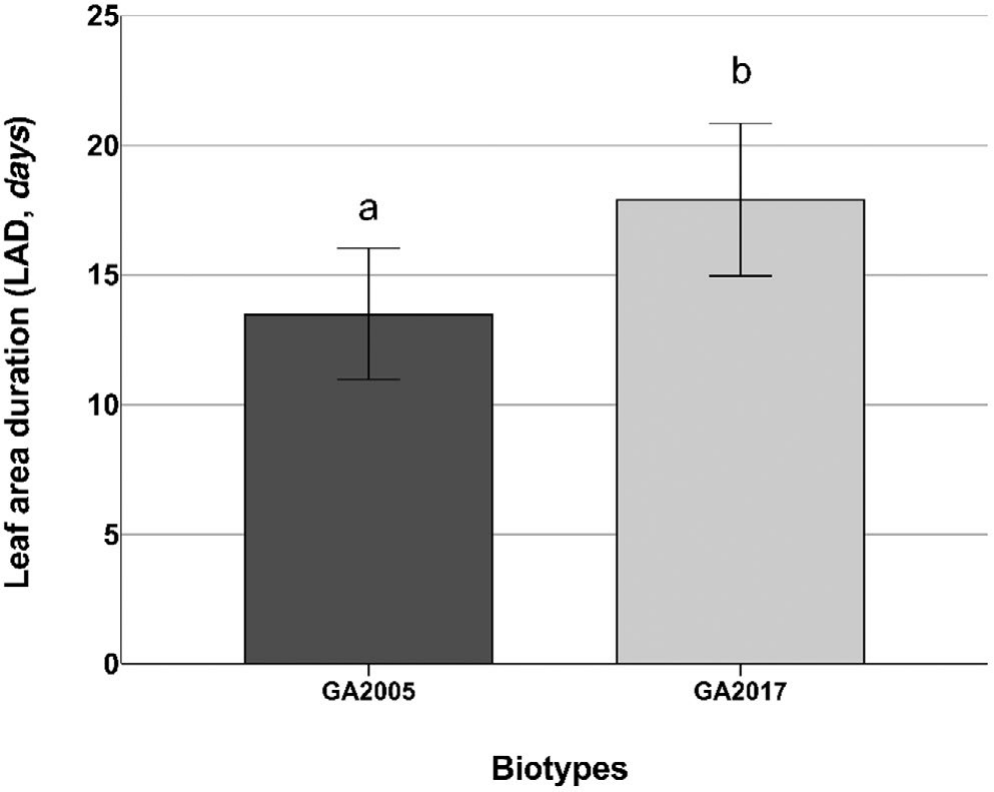
Main effects for leaf area duration (LAD, *days*) of *Amaranthus palmeri* biotypes, (GA2005 and GA2017). Data presented are means ± SE (standard error) from the original data. Means followed by the same letter are not different based on the Tukey HSD test at α = 0.05.

LAD exhibited statistical significance (*p* < 0.001) across harvest days (HD) and for biotypes (*p* = 0.0156) (Table 3). LAD remained stable up to 72 HD, averaging 13.4 days (about 2 weeks), followed by a notable increase at 102 HD, reaching 19.2 days (Figure 6). When comparing different biotypes, it was observed that the LAD was longer for GA2017, reaching approximately 17.9 days compared to 13.5 days for GA2005 (Figure 7).

**FIGURE 8:**
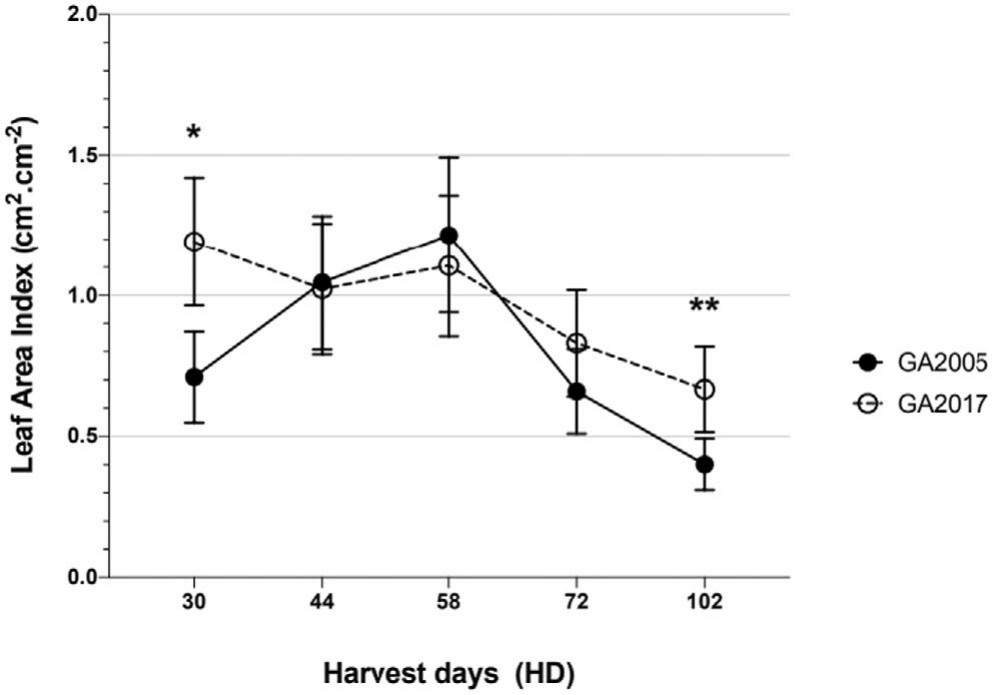
Interaction effects for leaf area index (LAI) of *Amaranthus palmeri* biotypes observed over harvest days (HD). The data presented shows biotype versus harvest days, with LAI greater at 30 and 102 HD, respectively, for GA2017 compared to GA2005. Error bars are means ± SE (standard error) of the original data. Signif. codes: ‘**’ 0.01; ‘*’ 0.05; *p* < 0.05.

Leaf area index (LAI), the rate of new leaf formation, and its persistence indicate a statistically significant interaction (*p* = 0.0485) between biotype and HD (Table 3). Pairwise comparisons demonstrated differences, with GA2017 having higher LAI at 30 (41.5%) and 102 (39.8%) HD compared to GA2005 (Figure 8).

## 4 Discussion

### 4.1 Shikimic Acid Accumulation

The results demonstrated that shikimic acid accumulation increased in response to higher glyphosate doses in both biotypes tested. Similar findings have been reported for other glyphosate‐resistant *A. palmeri* biotypes (Steckel et al. 2008; Whitaker et al. 2013; Dominguez‐Valenzuela et al. 2017). Shikimic acid accumulation has also been documented in glyphosate‐resistant weed populations such as *Lolium rigidum* (Fernández‐Moreno et al. 2016; Fernández‐Moreno, Bastida, and De Prado 2017), *L. multiflorum* (González‐Torralva et al. 2012), and *Eleusine indica* (Yu et al. 2015) when compared to susceptible populations. Glyphosate resistance in Georgia *A. palmeri* has been linked to EPSPS gene amplification, leading to an overexpression of the EPSPS enzyme (Gaines et al. 2011). This overexpression allows the plants to withstand herbicide treatments that would typically suppress their growth.

### 4.2 Growth Traits and Competitive Advantage

Research and interpretation of growth patterns not only elucidate how plants accumulate dry matter but also reveal the factors that can enhance or diminish a plant's productivity, whether individually or within a population (Zhang and Li 2019). These factors can include differences in how plants accumulate, use resources, and compete with neighboring plants (Kunstler et al. 2016). Intraspecific variability may explain how species can respond to biotic and abiotic stresses to maintain viable populations (De la Riva et al. 2016). Growth traits at the intraspecific level may vary even among a given species occurring in close locations, where a shift in resource use strategy can occur to adapt to the prevailing environmental conditions (Gagliardi et al. 2015).

Leaf weight ratio (LWR), leaf area ratio (LAR), specific leaf ratio (SLR), and relative growth rate (RGR) exhibited no variation among biotypes, only varying as a function of harvest days. However, distinctions in leaf area index (LAI), leaf area duration (LAD), and chlorophyll content were observed between the biotypes. Variability in growth and productivity is intimately tied to the amount of intercepted radiation, primarily defined by LAI, and the persistence of green tissue as captured by LAD. These indices are vital in ecophysiology, as they enhance gas exchange from the leaf level to the broader canopy scale (Bréda 2008). LAI is a crucial metric for estimating the leaf area within an ecosystem and is directly related to key processes such as photosynthesis, respiration, and precipitation interception (Alton 2016; Jarlan et al. 2008). Additionally, LAI regulates surface heat fluxes and transpiration rates, as larger leaves facilitate increased transpiration (Chase et al. 1996).

The glyphosate‐resistant biotype (GA2017) exhibited a higher LAI at the initial and final harvest stages, suggesting a potential adaptive advantage. In agricultural cropping systems, where competition for light, nutrients, and space is intense, a higher LAI could provide a substantial advantage to resistant biotypes, particularly during critical early development stages when crop‐weed competition is most significant. Such traits enable resistant populations to thrive under agricultural management practices that may otherwise suppress less adapted biotypes.

LAD complements LAI by providing a temporal dimension, integrating leaf area over time to reflect the duration of photosynthetic activity. This parameter highlights the cumulative capacity of a plant to intercept light, which is critical for biomass accumulation. In this study, the resistant biotype (GA2017) exhibited higher LAD, reflecting its ability to sustain a productive canopy for a longer period. Higher LAD and LAI may provide the resistant biotype (GA2017) with competitive advantages in resource acquisition and growth.

Chlorophyll content further influences this dynamic by enhancing the efficiency of light interception and energy conversion. In our research, the glyphosate‐resistant *A. palmeri* biotype (GA2017) exhibited higher chlorophyll content, which could stem from evolutionary pressures associated with herbicide resistance, differences in environmental conditions, or due to a growth strategy. Nonetheless, the precise factors driving changes in chlorophyll content remain unknown (Ryu, Berry, and Baldocchi 2019). Chlorophyll content plays a critical role in optimizing photosynthetic efficiency, particularly when LAI and LAD are already elevated. These findings suggest that the combination of higher LAI, LAD, and chlorophyll content would enable the resistant biotype evaluated in this study to improve competitiveness under agricultural conditions.

### 4.3 Genetic Background and Environmental Influence on Growth Traits

Genetic differentiation among *A. palmeri* populations is limited even at the regional level (Chandi, Milla‐Lewis, and Jordan 2013; Leon and van der Laat 2021). Although both biotypes were collected from close locations in Georgia, this research does not allow for definitive conclusions regarding the fitness cost associated with resistance because of the variable genetic background and traits related to the adaptation to cropping systems. The genetic constraints associated with glyphosate resistance, along with the unique microenvironments of specific locations, may influence the adaptations and growth of *A. palmeri*, as suggested by Bond and Oliver (2006) and Bravo et al. (2017). Their research indicates that variations in key morphological traits of *A. palmeri* are linked to cropping history. Bravo et al. (2017) proposed that glyphosate‐resistant populations could adapt more effectively to their cropping systems by developing greater height, increased biomass per plant, and an elongated canopy architecture compared to glyphosate‐susceptible populations. These adaptations, while not pleiotropic to glyphosate resistance, may help resistant populations to be more competitive than susceptible ones (Bravo et al. 2017, 2018).

Glyphosate resistance could be considered an adaptive trait that confers a fitness advantage in environments where glyphosate is used extensively (Haldane 1960). Resistance biotypes would be more likely to persist and spread when coupled with traits like higher LAI, chlorophyll content, and LAD. These traits provide a fitness advantage by enabling the biotypes to efficiently capture and utilize solar energy for growth and reproduction, making them a better fit and able to outcompete the susceptible biotypes under competition (Haldane 1960). However, to confirm whether there are fitness advantages to herbicide resistance, controlling for genetic background and competition studies under field conditions are necessary (Gressel 2005; Vila‐Aiub, Neve, and Powles 2009).

The observed differences in growth parameters—particularly leaf area index (LAI), chlorophyll content, and leaf area duration (LAD)—serve as an initial step, paving the way for future research opportunities to expand upon these identified growth parameters. Such research could clarify whether these factors significantly distinguish intraspecific growth differences associated with glyphosate‐resistant biotypes. Additionally, it may help us understand the mechanisms of species adaptation and the growth strategies that could further enhance their control.

## Conflicts of Interest

The authors declare no conflicts of interest.

## Data Availability

The data that support the findings of this study are openly available in OSF at http://doi.org/10.17605/OSF.IO/U8XGY. Reference: de Souza Rodrigues (2024).
